# Conditioned medium from asbestos-exposed fibroblasts affects proliferation and invasion of lung cancer cell lines

**DOI:** 10.1371/journal.pone.0222160

**Published:** 2019-09-06

**Authors:** Seunghye Yu, Hee-Hyun Choi, Il Won Kim, Tae-Jung Kim

**Affiliations:** 1 Department of Hospital Pathology, College of Medicine, The Catholic University of Korea, Seoul, Korea; 2 Department of Chemical Engineering, Soongsil University, Seoul, Korea; H. Lee Moffitt Cancer Center & Research Institute, UNITED STATES

## Abstract

The importance of the role of fibroblasts in cancer microenvironment is well-recognized. However, the relationship between fibroblasts and asbestos-induced lung cancer remains underexplored. To investigate the effect of the asbestos-related microenvironment on lung cancer progression, lung cancer cells (NCI-H358, Calu-3, and A549) were cultured in media derived from IMR-90 lung fibroblasts exposed to 50 mg/L asbestos (chrysotile, amosite, and crocidolite) for 24 h. The kinetics and migration of lung cancer cells in the presence of asbestos-exposed lung fibroblast media were monitored using a real-time cell analysis system. Proliferation and migration of A549 cells increased in the presence of media derived from asbestos-exposed lung fibroblasts than in the presence of media derived from normal lung fibroblasts. We observed no increase in proliferation and migration in lung cancer cells cultured in asbestos-exposed lung cancer cell medium. In contrast, increased proliferation and migration in lung cancer cells exposed to media from asbestos-exposed lung fibroblasts was observed for all types of asbestos. Media derived from lung fibroblasts exposed to other stressors, such as hydrogen peroxide and UV radiation didn’t show as similar effect as asbestos exposure. An enzyme-linked immunosorbent assay (ELISA)-based cytokine array identified interleukin (IL)-6 and IL-8, which show pleiotropic regulatory effects on lung cancer cells, to be specifically produced in higher amounts by the three types of asbestos-exposed lung fibroblasts than normal lung fibroblasts. Thus, the present study demonstrated that interaction of lung fibroblasts with asbestos may support the growth and metastasis of lung cancer cells and that chrysotile exposure can lead to lung cancer similar to that caused by amphibole asbestos (amosite and crocidolite).

## Introduction

Lung cancer, one of the respiratory diseases caused by asbestos inhalation, is estimated to cause higher annual deaths than other asbestos-related diseases. Asbestos-induced lung cancer is further aggravated by pulmonary fibrosis, which provides a favorable environment for lung cancer development [1]. Indeed, radiographic and histological evidence shows that most patients with lung cancer employed in the asbestos cement and asbestos insulation industries were affected by pulmonary fibrosis. These reports demonstrated that excessive asbestos can act as a lung carcinogen because of its fibrogenicity [2].

Fibroblasts, the end effector cells of fibrosis in fibrotic lungs, are embedded within the interstitium of all epithelial tissues and play important roles in organogenesis, wound healing, inflammation, and fibrosis [3]. In particular, fibroblasts that have acquired an activated phenotype (activated fibroblasts and cancer-associated fibroblasts), characterized by the expression of α-smooth-muscle actin (α-SMA) and secretion of increased amounts of extracellular matrix (ECM) components and growth factors such as transforming growth factors-β (TGF-β), promote tumor growth and progression. These types of fibroblasts are often referred to as myofibroblasts because of the expression of α-SMA, a myofibroblast marker [4].

Asbestos fibers deposited in interstitial spaces are phagocytosed by macrophages and epithelial cells [5, 6], which subsequently alter the morphology and biochemistry of fibroblasts during fibrogenesis [7]. As myofibroblasts are the predominant sources of collagen and fibrogenic cytokines in fibrotic lesions, previous studies showing that direct exposure of lung fibroblasts to asbestos increases deposition of collagen or ECM constituents, including hydroxyproline [8], have postulated that asbestos-treated lung fibroblasts have the potential to activate or differentiate into myofibroblasts and consequently cause fibrosis [9].

These fibroblasts also continually modify their interactions with the lung microenvironment and are capable of supporting the dynamic complexity of tumor microenvironment [10]. For example, the secretory functions of activated fibroblasts positively mediate proliferation, survival, and metastasis of cancer cells via cell-cell contact or paracrine/exocrine signaling at the secondary tumor growth or metastatic sites [4]. Also, activated fibroblasts promote angiogenesis, which indirectly affects the migratory and invasive properties of cancer cells [11]. Therefore, activated fibroblasts or myofibroblasts are accepted niches for understanding the mechanisms of asbestos-induced lung cancers as they can act as physical supporting elements as well as regulatory components [12] for cancer growth and metastasis [13] in the lung microenvironment [14].

Despite the possibility that the asbestos-induced microenvironment might increase the growth and metastatic potential of lung cancer [15], a detailed mechanism correlating carcinogenesis with the recruitment of asbestos-exposed fibroblasts, myofibroblasts, or fibrosis is still being unraveled. To better understand the kinetics of the process via which asbestos-exposed lung fibroblasts affect lung cancer development, we treated lung cancer cells with asbestos-exposed fibroblast-derived media. Real-time cell analysis (RTCA) platforms, which are currently being used [16] for their real-time and label-free analytical ability, can assess cell growth and migratory kinetics. This system can recognize the attachment of adherent cells as electrode impedance and measure cellular response, including proliferation and migration in physiologically relevant conditions.

The purpose of this study was to investigate the effects of different types of asbestos-exposed lung fibroblasts on lung cancer cells. Using the RTCA system, we assessed the response of lung cancer cells to media derived from lung fibroblasts exposed to three representative types of asbestos, chrysotile, amosite, and crocidolite. We also demonstrated that common cytokines were responsible for the stimulation by asbestos-exposed lung fibroblast-derived media.

## Materials and methods

### Asbestos samples

Three types of Union Internationale Contre le Cancer (UICC) standard asbestos (chrysotile #02701-AB, amosite #02703-AB and crocidolite #02704-AB) were purchased from SPI supplies (West Chester, PA USA). The samples were suspended in deionized water (18.2 MΩ·cm, Direct-Q, Millipore, Billerica, MA, USA, #ZR0Q00800) and sonicated in an ultrasonic cleaner (BRANSON 8510, Branson, Danbury, CT, USA, #B8510E-MTH) for 2 h.

### Asbestos morphology

The shapes of the fibers were determined using scanning electron microscopy (SEM: GeminiSEM 300, Carl Zeiss, Oberkochen, Germany) as shown in S1 Fig. The sample was Au-coated using sputter-coater Quorum Q150R S (Quorum Technologies Ltd., Lewes, UK) prior to SEM observation.

### Asbestos crystallinity

The crystalline information (S2 Fig) of asbestos was obtained using the solid residues and an X-ray diffractometer (XRD: D2 PHASER, Bruker Corp., Billerica, MA, USA) equipped with a CuKα radiation source of λ = 0.15418 nm (10 mA and 30 kV). The data were obtained by scanning the 2θ region between 7° and 80° at a scan rate of 1.0°/min.

### Cell culture

Human lung cancer cell lines (NCI-H358, **ATCC** ® CRL-5807, lung bronchioloalveolar carcinoma; Calu-3, **ATCC** ® HTB-55, lung adenocarcinoma; A549, **ATCC** ® CCL-185, lung adenocarcinoma) and IMR-90 human lung fibroblast cell lines (**ATCC** ® CCL-186) were obtained from the American Type Culture Collection (ATCC, Rockville, MD, USA). IMR-90 and Calu-3 cells were cultured in Eagle’s minimal essential medium (EMEM), NCI-H358 cells in Roswell Park Memorial Institute (RPMI)-1640 medium, and A549 cells in F-12K medium supplemented with 10% fetal bovine serum (FBS, WelGENE Inc., Deagu, South Korea) and 100 U/mL penicillin-streptomycin (WelGENE Inc.) in a humidified incubator set at 37°C with 5% CO_2_. Subsequently, NCI-H358 and A549 cells were cultured in EMEM to match the medium used for IMR-90 cells. We performed cytotoxicity and cell viability assays as well as real-time cell monitoring for 120 hours to observed the effects of exposure to 50 mg/L chrysotile, 50 mg/L crocidolite, and 50 mg/L amosite on IMR-90 making IMR-90 being cytostatic, and decided to use 50mg/L as our experimental concentration for all types of asbestos [17]. The study was approved by the institutional review board of Yeouido St. Mary’s Hospital (Korea; approval number SC19ZNSI0015) and was in accordance with the relevant legislation.

### Confocal laser microscope

We used a confocal laser microscope to observe how lung fibroblasts gained myofibroblastic features by expressing increased cytoplasmic actin filaments. Briefly, IMR-90 cells (50,000 cells/well) were plated in four-well chamber slides (#154526; Thermo Fisher Scientific^™^, Waltham, MA, USA) and treated with 50 mg/L chrysotile, amosite, or crocidolite for 1, 12, and 24 hours. After incubation, the cells were treated with 200 nmol/L MitoTracker^™^ Red (Molecular Probes/Invitrogen, Carlsbad, CA, USA) for 25 min at 37°C and 5% CO_2_, fixed using the Image-iT^®^ fixation/permeabilization kit (Molecular Probes), washed three times with PBS, and non-specific sites were blocked with PBS containing 2 g/L bovine serum albumin for 60 min at 25°C. Cells were then stained with 200 μL of 3× Dulbecco’s PBS (DPBS) containing 5 μL of Alexa Fluor^®^ 488 phalloidin (200 units/mL in methanol; Molecular Probes) for 20 min and washed with DPBS; nuclei were counterstained with NucBlue^®^ Fixed Cell ReadyProbes^®^ Reagent (R37606; Molecular Probes/Invitrogen). The slides were covered with coverslips and cells were observed and photographed under a confocal laser scanning microscope (LSM 710; Carl Zeiss, Jena, Germany). Images were processed using the Image Examiner software (Carl Zeiss). Confocal laser microscopy showed an increase in actin filaments with preserved mitochondrial activity in IMR-90 cells after 24 hours of exposure to each asbestos fiber. After 48 hours of exposure to asbestos fibers, IMR-90 cells showed increased actin filaments but decreased or clumped mitochondrial activity (S3 Fig). Hence, we decided to measure the effect on IMR-90 cells with myofibroblastic features after 12 hours of exposure to asbestos fibers.

### Asbestos-exposed cell-derived media

For preparing the media of asbestos-exposed lung fibroblast and cancer cells, 50,000 cells/mL of lung cancer cells and IMR-90 cells were seeded into 0.4 μm transwell polycarbonate membranes (costar 3401, Corning, West Chester, PA, USA), and cultured for 24 h with or without 50 mg/L of the three types of asbestos (chrysotile, amosite, and crocidolite). Then, the media were aspirated from the wells and used in experiments mentioned in the following sections.

### Cell proliferation

Cell proliferation was analyzed in real-time using the commercially available impedance-based xCELLigence System (Roche Applied Science, Mannheim, Germany) in E-plates 16 (ACEA Biosciences, Inc) according to the manufacturer's instructions. The background signal of the culture medium was the first set up in an E-plate 16. Lung cancer cells were seeded in E-plates per the cell number obtained after titration in lung fibroblast cell-derived media (S4 Fig); the cell numbers were 10,000 cells/well for NCI-H358, 40,000 cells/well for Calu-3, and 4,000 cells/well for A549. The signal was measured every 15 min, starting immediately after the seeding. Twenty-four hours after seeding, the culture media were removed and replaced with the asbestos-exposed lung fibroblast-derived media and asbestos-exposed lung cancer cell-derived media. Cell groups directly exposed to asbestos were also included. Normalized cell index (NCI) was calculated by dividing every CI at any given time by CI at the normalized point. The experiment was performed in triplicate or quadruplet.

### Cell migration

The migration characteristics of the A549 lung cancer cell line was investigated by installing CIM-plate 16 in RTCA, which allowed migration of cells toward the chemoattractant side through the microporous wall membrane. Media from IMR-90 and A549 cells and 50 mg/L of asbestos-exposed IMR-90 and A549 cells were added to the lower chamber (LC) of the CIM-plate 16. Media (EMEM) with or without 50 mg/L of asbestos were also placed in the LC. Two-hour serum-starved A549 cells were plated on the upper chamber (UC) of the CIM-plate 16 at a density of 20,000 cells/well with serum-free media. The effects of the tested media on A549 cells that pass through the electrodes from the UC to the LC are shown as CI values. This experiment lasted for 24 h.

### Assessing cell proliferation after H_2_O_2_ and UV treatment

Preliminary experiments were conducted on CellTiter-Glo to determine the treatment conditions for hydrogen peroxide (H_2_O_2_, Duksan Pure Chemicals, Gyeonggi-do, Korea) and UV radiation (UV crosslinker. Fisher Biotech, Pittsburgh, PA, USA) such that survival rates similar to those of IMR-90 cells exposed to 50 mg/L asbestos for 24 h were obtained (S5 Fig). IMR-90 cells were plated on a 24-well plate at a density of 50,000 cells/mL. After incubation, the cells were covered with DPBS and exposed to 1 mM H_2_O_2_ for 3 h or 25 J/m^2^ UV radiation. The damaged cells were immediately incubated in fresh media for 24 h. To monitor cell response using RTCA, NCI-H358 (10,000 cells/well), Calu-3 (40,000 cells/well), and A549 (4,000 cells/well) cells were seeded on E-plate 16. Following 24 h, the media were removed and replaced with EMEM, IMR-90 cell-derived media, and media derived from IMR-90 cells treated with H_2_O_2_ and UV.

### Analysis of secretome content

Asbestos-exposed IMR-90 cell-derived media were obtained by collecting the targeted medium as described above. The presence of common cytokines and chemokines (interleukin (IL)-1α, IL-1β, IL-2, IL-4, IL-5, IL-6, IL-8, IL-10, IL-12, IL-13, IL-17A, and granulocyte-macrophage colony-stimulating factor (GM-CSF)) in the media was determined using the Multi-Analyte ELISArray kit (#MEH-0006A, Qiagen). Briefly, the collected cell culture supernatants were placed on the kit’s plate and incubated for 2 h at room temperature. After washing the membrane to remove unbound protein, biotinylated detection antibodies were added to bind to the analyte. After incubating at room temperature for 1 h and washing, an avidin-horseradish peroxidase conjugate was added and incubated for 30 min at room temperature. A colorimetric substrate solution that can directly measure the amount of protein present in the sample was added and incubated for 15 min, followed by addition of the stop solution and measurement of absorbance at 570 and 450 nm using an ELISA reader (Power wave XS, BioTek, VT, USA).

### Statistical analysis

Statistical analysis was performed using the one-way analysis of variance for more than three samples and Student’s t-test for comparison of two samples (SPSS.v21 Statistical Package, SPSS Inc., Chicago, IL, USA). Data were analyzed as mean ± standard deviation (SD) of at least three independent experiments. Probability (P) values < 0.05 were considered significant.

## Results

### Dynamic real-time cellular profiles of asbestos-exposed lung fibroblast-derived media in lung cancer cells

Real-time electronic cell sensor arrays were used to monitor the behavior of lung cancer cells in the presence of media derived from asbestos-exposed IMR-90 lung fibroblast cells. Various types of media were designed: IMRM (IMR-90 cells-derived media), IMRM-C (50 mg/L chrysotile-exposed IMR-90 cells-derived media), IMRM-A (50 mg/L amosite-exposed IMR-90 cells-derived media), and IMRM-D (50 mg/L crocidolite-exposed IMR-90 cells-derived media). In the same manner, media from three types of lung cancer cells have been abbreviated as follows: NCI-H358; NCIM (NCI-H358 cells-derived media), NCIM-C (50 mg/L chrysotile-exposed NCI-H358 cells-derived media), NCIM-A (50 mg/L amosite-exposed NCI-H358 cells-derived media), NCIM-D (50 mg/L crocidolite-exposed NCI-H358 cells-derived media), Calu-3; CALM (Calu-3 cells-derived media), CALM-C (50 mg/L chrysotile-exposed Calu-3 cells-derived media), CALM-A (50 mg/L amosite-exposed Calu-3 cells-derived media), CALM-D (50 mg/L crocidolite-exposed Calu-3 cells-derived media), A549; A549M (A549 cells-derived media), A549M-C (50 μg/mL chrysotile-exposed A549 cells-derived media), A549M-A (50 mg/L amosite-exposed A549 cells-derived media), and A549M-D (50 mg/L crocidolite-exposed A549 cells-derived media).

**Fig 1:**
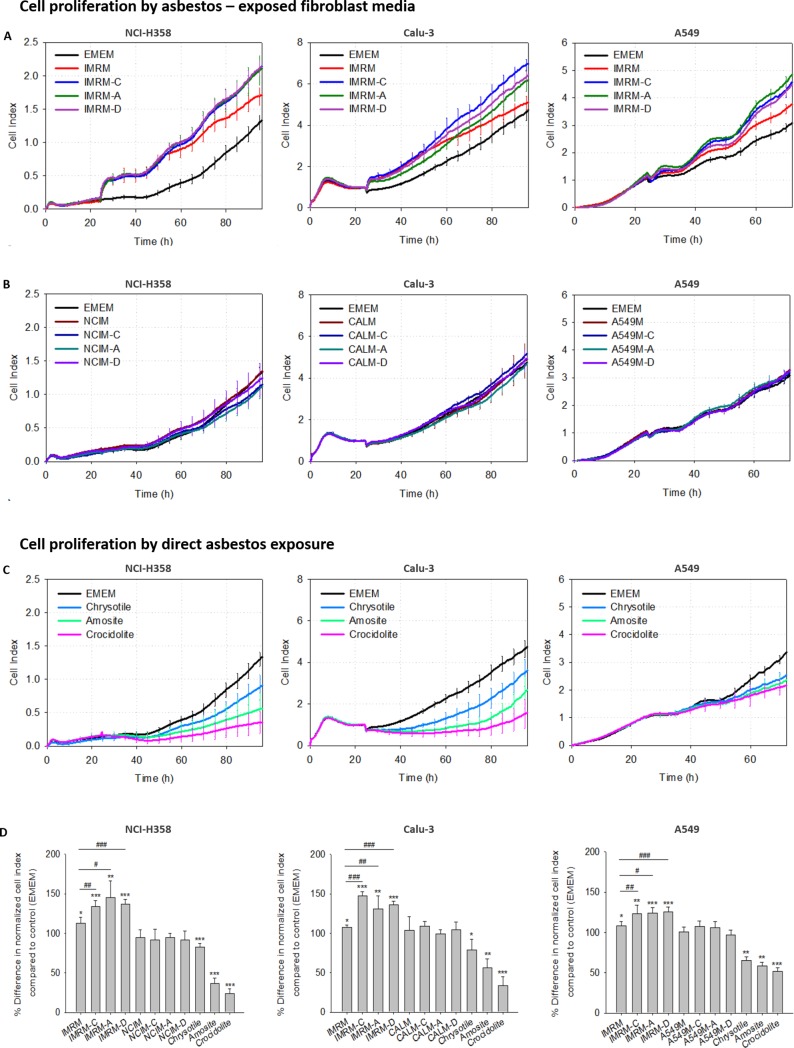
RTCA traces showing the effect of three types of asbestos-related media on NCI-H358, Calu-3, A549 lung cancer cells. Following 24 h of seeding, NCI-H358, Calu-3 cells, A549 cells were cultured with derived media from (A) IMRM, IMRM-C, IMRM-A, and IMRM-D, (B) NCIM, NCIM-C, NCIM-A, NCIM-D, CALM, CALM-C, CALM-A, CALM-D, A549M, A549M-C, A549M-A, and A549M-D (C) direct exposure to 50 mg/L of three types of asbestos (chrysotile, amosite, and crocidolite). Cells were also grown in EMEM normal medium. (D) Comparison of the percentage difference in the mean NCI compared to control (EMEM) at 96 h. Error bars represent SD (n = 4). *P < 0.05, **P < 0.01, ***P < 0.001 compared to EMEM, and #P < 0.05, ##P < 0.01, ###P < 0.001 for comparisons between two groups.

When NCI-H358, Calu-3, and A549 were exposed to IMRM, IMRM-C, IMRM-A, and IMRM-D for 24 h in the experimental period, the growth of all three lung cancer cell lines increased promptly. At any given time point, NCI-H358, Calu-3 and A549 cells incubated in IMRM, IMRM-C, IMRM-A, and IMRM-D proliferated more than in those incubated with EMEM (P < 0.05, n = 4). Importantly, after approximately 30 h, cell growth was higher in IMRM-C, IMRM-A, and IMRM-D than in IMRM (Fig 1A). On the other hand, when lung cancer cells were grown in the presence of each lung cancer cell-derived media or asbestos-exposed lung cancer cell-derived media, the lung cancer cells displayed no difference in growth rates and did not proliferate compared to those grown in EMEM (Fig 1B). The growth of all lung cancer cells was significantly inhibited when exposed directly to asbestos (Fig 1C) (P < 0.05, n = 4). Statistical analysis of NCI value against the control (EMEM) indicated significant differences among various derived media at 96 h (NCI-H358 and Calu-3 cells) and 72 h (A549 cells) (Fig 1D). These studies demonstrated that direct exposure of lung cancer cells to asbestos induces cytotoxic effects, whereas the interaction between asbestos and lung fibroblasts favored the growth of lung cancer cells.

### Monitoring cell migration behavior in real time

**Fig 2:**
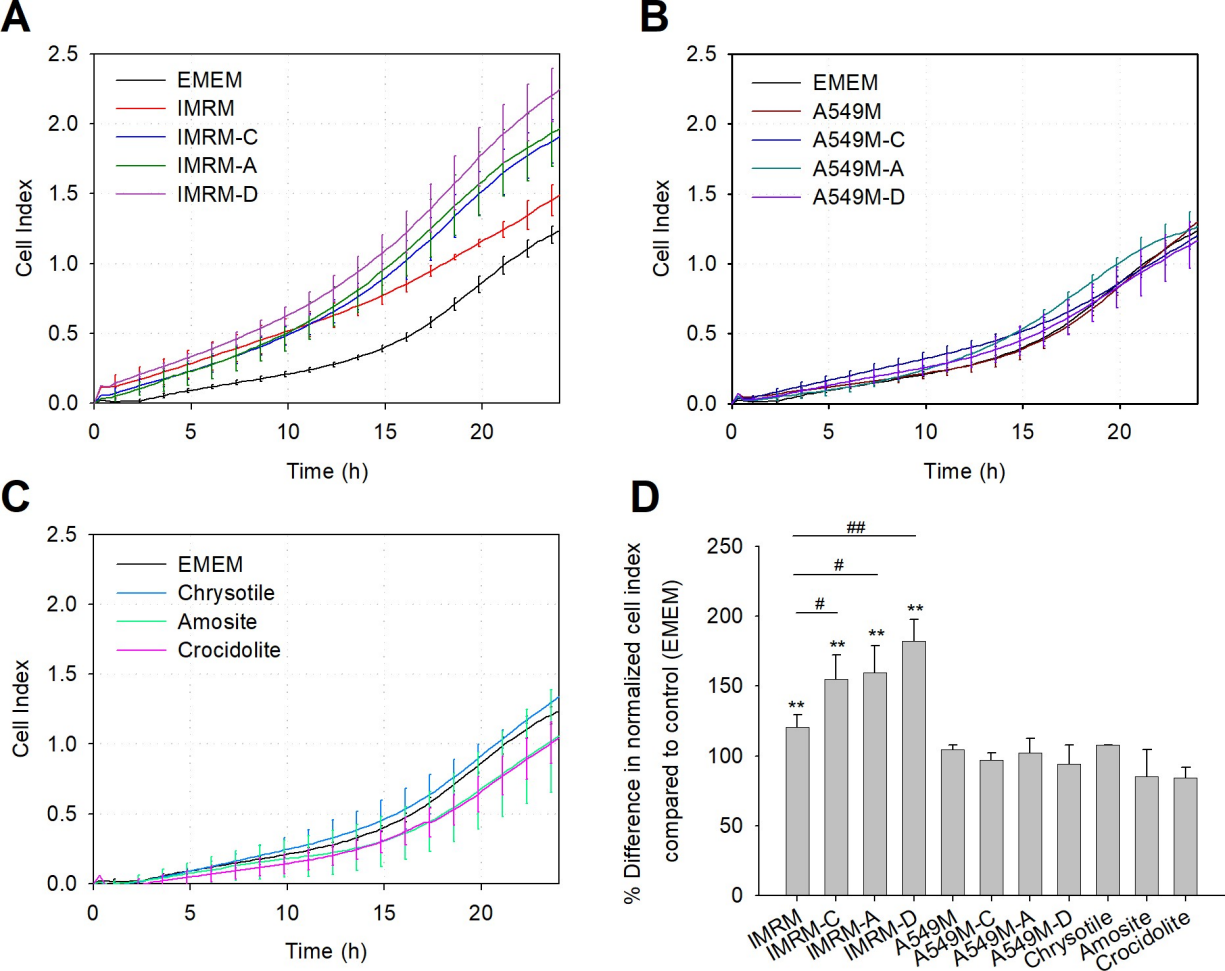
Monitoring the migration of A549 cells. Migration of A549 cells in various media were monitored for 24 h using the RTCA system. Representative graph of migration of A549 cells towards (A) IMRM, IMRM-C, IMRM-A, IMRM-D, and EMEM, (B) A549M, A549M-C, A549M-A, A549M-D, and EMEM, (C) chrysotile, amosite, crocidolite, and EMEM. (D) Comparison of calculated NCI values of migration in all media at 24 h. Experiments were performed in quadruplet and presented as mean ± SD. Quantification of observed migration revealed significance., **P < 0.01 compared to EMEM and #P < 0.05, ##P < 0.01 compared to IMRM.

The rate of migration of lung cancer cells in the presence of media obtained from asbestos-exposed lung fibroblasts was assessed (Fig 2). Fibroblasts were assessed the UC of CIM-plate 16, while the target media was placed in the LC of CIM-plate 16, and the migration of these cells was monitored over 24 h. The optimal cell density for assessing cell migration was 20,000 cells/well for A549 cells; NCI-H358 and Calu-3 cells could not be used for migration assays due to lack of migration affinity in the RTCA system (S6 Fig). The evolution of the CI value with time, which indicated the migration rate of A549 cells, showed that the rate of migration to IMRM was higher than that of EMEM (P < 0.05, n = 4). More importantly, A549 cells migrate more rapidly toward IMRM-C, IMRM-A, and IMRM-D than to IMRM (P < 0.05, n = 4) (Fig 2A and 2D). In contrast, A549M, A549M-C, A549M-A, A549M-D, and direct addition of asbestos in LC did not enhance migration over time (Fig 2B, 2C and 2D). These results indicate the synergistic effect of the interaction between asbestos and lung fibroblast cells on lung cancer cell migration.

### Kinetics of lung cancer cell proliferation in the presence of media derived from stress-induced lung fibroblasts

**Fig 3:**
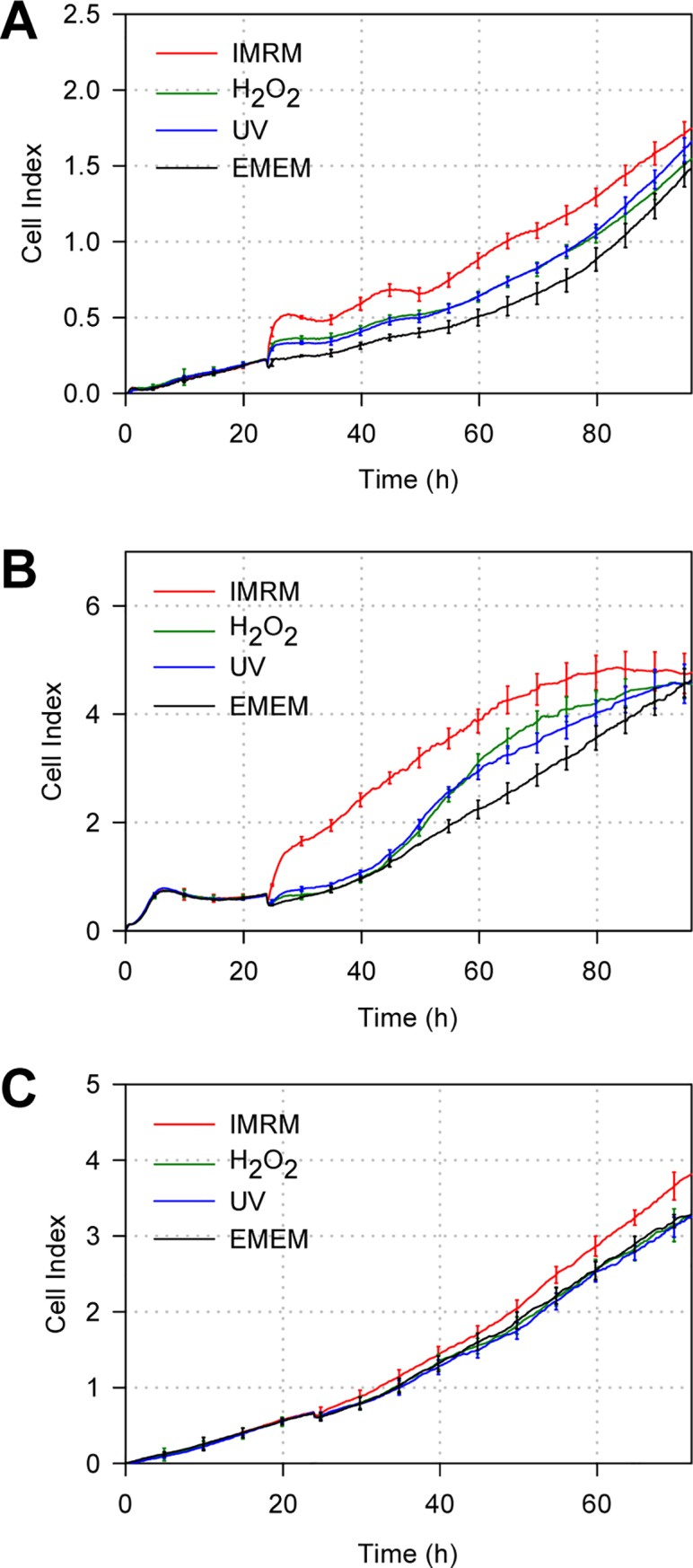
Effects of media derived from H_2_O_2_ and UV-stressed lung fibroblasts on lung cancer cells. (A) RTCA sensing profile of CI values over time for (A) NCI-H358, (B) Calu-3, and (C) A549 cells exposed to H_2_O_2_ or UV-treated IMR-90 cell-derived media. The experiment was also conducted in EMEM and IMRM as controls. Data show mean ± SD (n = 3).

To compare the stress induced by asbestos with those induced by other stressors, two most widely used stress inducers (H_2_O_2_ and UV radiation) were tested on lung cancer cells (Fig 3). Media derived from IMR-90 cells following exposure to H_2_O_2_ and UV appeared to be less potent in enhancing the growth of NCI-H358 (Fig 3A), Calu-3 (Fig 3B), and A549 (Fig 3C) cells. The cultures were grown in media derived from H_2_O_2_- and UV-exposed IMR-90 cells showed either similar growth rates compared to those of EMEM or lower values than those of IMRM.

### Analysis of cytokine profiles after asbestos exposure

**Fig 4:**
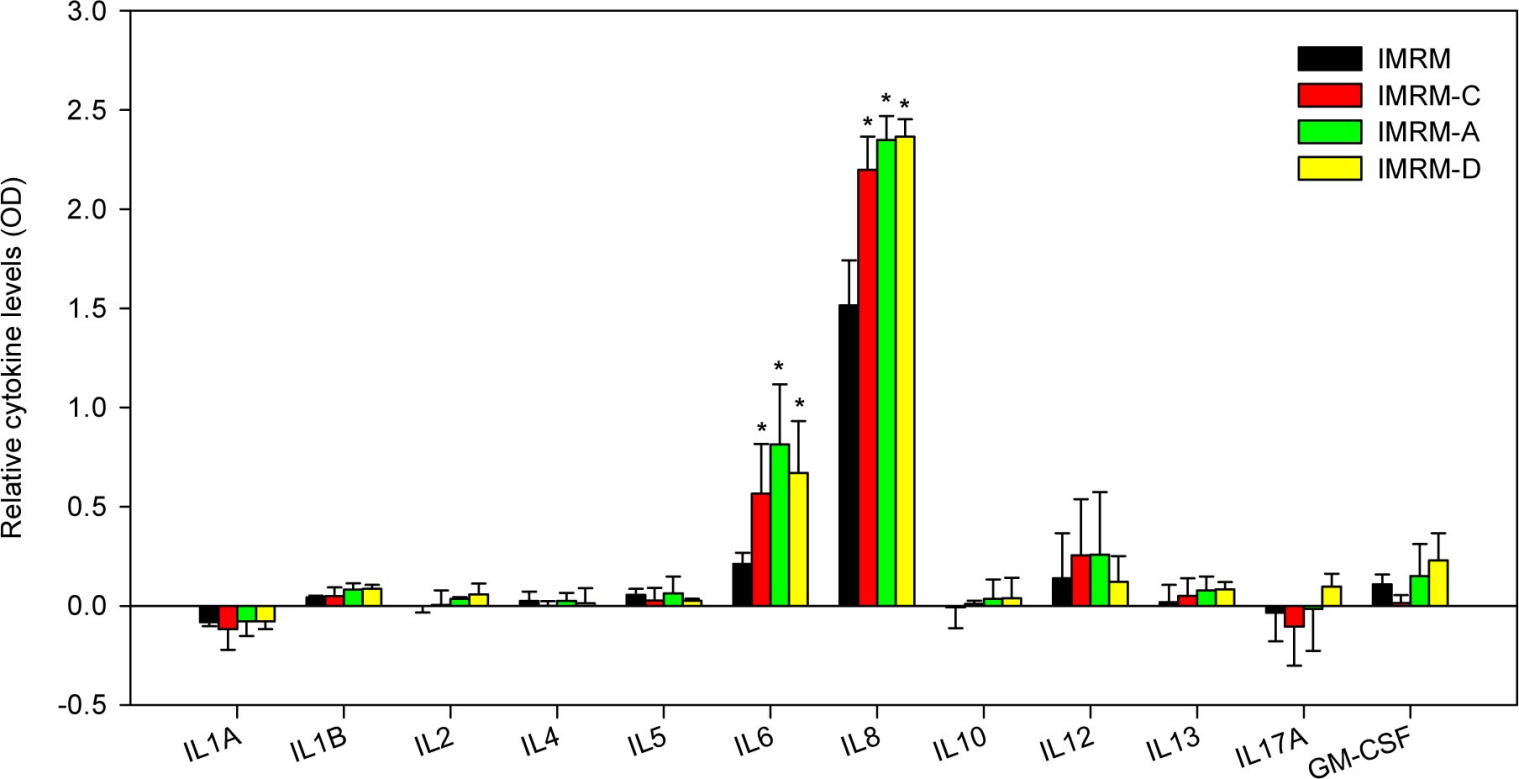
Asbestos-exposed IMR-90 cells show increased cytokine secretion. Cytokines in the supernatants of IMR-90 cells exposed or unexposed to asbestos for 24 h were measured using ELISA. The results are expressed as mean ± SD after subtracting negative value from each value. *P < 0.05, vs. IMRM group, n = 4.

We next investigated the cytokine profile of asbestos-exposed IMR-90 cells at the protein level using an enzyme-linked immunosorbent assay (ELISA). Supernatants of IMR-90 cells treated with or without 50 mg/L of asbestos for 24 h were prepared and used against a panel of 12 common cytokines (IL-1α, IL-1β, IL-2, IL-4, IL-6, IL-8, IL-10, IL-12, IL-13, IL-17A, and GM-CSF). Among the cytokines tested, IL-6 and IL-8 were considerably induced upon exposure to IMRM-C, IMRM-A, and IMRM-D compared to IMRM (Fig 4). The cytokine analysis revealed that although IMR-90 cells themselves secrete IL-6 and IL-8, asbestos exposure induced even higher levels of both IL-6 and IL-8 than IMRM. Supernatants of IMR-90 cells showed similar levels of IL-6 and IL-8 secretion for all types of asbestos tested.

## Discussion

Although the association between asbestos exposure and an increased risk of bronchogenic carcinoma is indisputable [18], few studies have considered the effects of lung fibroblasts in this process. Recent studies regarding tumor microenvironment clearly showed that activated fibroblasts and myofibroblasts surrounding the cancer cells are associated with cancer growth and metastasis [13, 19]. Therefore, lung fibroblasts, present at the active site of pulmonary fibrosis in the interstitium where asbestos fibers embed, are as likely to be associated with lung cancer development as asbestos itself [20]. Therefore, it is important to investigate the interactions between asbestos and lung fibroblasts and how is affects lung cancer cells to obtain new insights regarding asbestos-induced lung cancer. There is a strong argument regarding the relationship between asbestos exposure and lung cancer due to the distribution of the related conditions. Asbestosis is primarily a disease of the lung periphery that is typically more severe in the lower lobes, whereas asbestos-related bronchogenic carcinoma is a more central process that commonly affects the upper lobes [21].

In this study, lung cancer cells were cultured with asbestos-exposed IMR-90 lung fibroblast-derived media to establish how paracrine signaling, which has a potential impact on carcinogenesis despite the anatomic distance, play a role as asbestos related tumor microenvironment. A previous study used an experimental design similar to that of our study to describe tumor microenvironment [22]. This experimental method allows to identify the characteristics of the interaction between cancer cells and fibroblasts such as cancer proliferation, promotion, and toxicity, analyze the genes involved using conditioned media *in vitro*, and study the tumor microenvironment. This is a practical technique that serves as a basis for studying the function of target genes, which may later affect the growth of cancer cells in the tumor microenvironment.

We established an asbestos-induced lung microenvironment by culturing lung cancer cells with media derived from asbestos-exposed IMR-90 lung fibroblast cells and monitored how asbestos-exposed lung fibroblasts modify the proliferation and migratory properties of lung cancer cells utilizing the RTCA system. RTCA overcomes the limitations of the endpoint assay and facilitates measurement of cellular perturbation such as a number of attached cells and cell morphology that respond to the environment in real-time. The concentration of asbestos was set to ensure asbestos-related intracellular toxicity according to the results of a previous study on the effect of various types of asbestos in cells [17].

IMR-90 lung fibroblast-derived media increased the growth of NCI-H358, Calu-3, and A549 lung cancer cells compared to fresh EMEM with 10% FBS and 100 U/mL penicillin-streptomycin. Previously published data on human dermal fibroblasts and human colon cancer cells indicated that fibroblasts possess the ability to stimulate the proliferation and migratory properties of cancer cells [13, 23, 24]. However, more importantly, when lung cancer cells were grown in the presence of media derived from asbestos-exposed lung fibroblast cells, all the lung cancer cell lines exhibited increased growth rate than those grown in IMR-90 cell-derived media (Fig 1). The characteristics of cellular and tissue reactions after a brief asbestos exposure have been previously defined *in vivo* [25]. In that study, the authors observed that inhaled asbestos fibers were almost exclusively located in the interstitium of the model rats. In particular, the asbestos fibers were located in fibroblasts, and this was accompanied by increased areas of myofibroblast/smooth muscle cell aggregates. Additional report on the enhanced production of fibrous collagen in cultures of rabbit lung fibroblasts with 50 mg/L asbestos indicate the fibrogenic potential of asbestos-exposed fibroblasts that may contribute to the progression of pulmonary fibrosis [26]. In this way, activated fibroblasts and myofibroblasts, which are not eliminated by apoptosis, play crucial roles in promoting carcinogenesis by secreting various factors [27].

In striking contrast, asbestos-exposed lung cancer cell-derived media did not enhance proliferation of lung cancer cells and direct exposure of lung cancer cells to asbestos was cytotoxic. This toxic effect has previously been reported as a part of the cellular response to asbestos-associated lung cancers. Indeed, apoptosis of A549 cells was induced by chrysotile asbestos exposure via the activation of c-Jun N-terminal kinase (JNK), which is linked to lung diseases [28]. Nevertheless, as signals from IMR-90 cells cultured with asbestos also provided an attractive gradient for faster migration of A549 cells as well as the proliferation of lung cancer cells, direct asbestos-lung fibroblast contact has implications in the pathophysiology of asbestos-induced lung cancers. This result suggests that the effect of fibroblasts exposed to asbestos on cancer cell migration is potentially synergistic, especially on A549 cells. Tumor progression not only depends on the cells type but on aberrant mutations or gene dysregulation [29]. Therefore, it would be interesting to study the timing of the effects of fibroblast-derived media on other cell lines.

In addition, our observation that lung cancer cells rarely proliferate when cultured in the presence of media from IMR-90 cells stressed by H_2_O_2_ and UV indicates that asbestos exposure promotes the growth of lung cancer cells distinctively unlike the stimulation by other stress inducers (Fig 3).

Previous studies have shown that cancer-associated fibroblasts (CAF) secrete a variety of cytokines in and high levels of growth factors such as EGF, TGF-β, HGF, and FGF-2 into CAF-conditioned media [30]. Thus, we selected the cytokine panel to identify the effects of CAF-derived genes on cancer cells and analyzed and quantified the cytokines secreted to the culture medium.

We, therefore, analyzed and quantified cytokines secreted in culture supernatants for further understanding the underlying mechanism of action. The secreted cytokines were tethered, biotinylated, and detected via a series of antigen-antibody reactions using the Multi-Analyte ELISA array. Results demonstrated that the proinflammatory cytokines IL-6 and IL-8 were markedly released from IMR-90 cells upon exposure to all three types of asbestos (Fig 4). Production of IL-6 and IL-8 affect the growth and migration of lung cancer cells as it plays a potential role in cancer development. For instance, several investigations have shown that secretion of IL-6 induces survival and proliferation of cancer cells and accelerates tumor growth via paracrine [31] and autocrine signaling [32]. Furthermore, it functions as a mediator that activates cancer cells against apoptosis via mitogen-activated protein kinase (MAPK) signaling or signal transducer and activator of transcription (STAT) factor [33]. The activation of jak2 / stat3 by IL-6 overexpression increased tumor initiation and affected the levels of many kinases in A549 and CL1-5 lung cancer cells. Blockade of the IL-6 / JAK2 / STAT3 pathway reduced the ability of A549 and CL1-5 cells to form tumors [34, 35]. IL-6 deficiency diminished the migration and invasion-promoting effects of adipose stromal cells as well as lung cancer cells [36].

Therefore, blockade of IL-6 signaling may act as an effective treatment for lung cancer [31]. IL-8 is also released in response to various external factors. The various stimuli, including inflammatory signals, biological species, and environmental stress enhance IL-8 production via the NF-κB pathway [37]. It also activates MAPK p42/44 downstream signaling pathways, resulting in the proliferation and survival of lung cancer cells [38]. In addition, IL-8 released from lung fibroblasts acts as an angiogenic inducer and regulator of the survival and growth of cancer cells [39, 40]. Taken together, the release of IL-6 and IL-8 from IMR-90 cells as a consequence of asbestos exposure can be highlighted as one of the possible carcinogenesis. However, more precise understanding of the molecular mechanism is required to understand whether these factors induce the growth and migration of cancer cells or whether additional interactions with other substances are also required [38].

Our observations also suggest that chrysotile asbestos, which is softer and less dangerous than the amphibole asbestos (amosite and crocidolite), possesses pathogenic potential [41]. Previous studies have postulated that amphibole asbestos is durable and strong owing to its structure, which includes a double chain of tetrahedral silicate with silica, whereas chrysotile favorably dissolves in tissues because of the presence of magnesium on its exterior [42, 43]. Nonetheless, this study shows that interaction between chrysotile and lung fibroblasts in the lung microenvironment might induce lung cancer development as much as amphibole asbestos.

Molecular studies have shown that chrysotile-exposed-human mesothelial cells are more adhesive to tissue culture dishes and express and release higher levels of TNF-α growth factors than cells exposed to other asbestos [44]. Chrysotile is less toxic than crocidolite and amosite but induces the expression of more inflammatory genes related to cancer progression [17]. Taken together, these results suggest that the interaction between chrysotile and lung fibroblasts in the tumor microenvironment can promote lung cancer development as much as the exposure to amphibole asbestos (amosite and crocidolite).

Our study proposes a simplified model to identify the interactions between tumor and surrounding cells and focusses on the characteristics of cancer and surrounding cells and the genes associated with asbestos-exposed fibroblasts. Therefore, it should be noted that the complexity of the critical microenvironment is excluded.

## Conclusions

In summary, our study shows that the progression of asbestos-related lung cancer is substantially associated with asbestos-exposed lung fibroblasts *in vitro*. Growth and migration of lung cancer cells were obviously promoted in the presence of media derived from three types of asbestos (chrysotile, amosite, and crocidolite)-exposed lung fibroblasts, which contained high levels of IL-6 and IL-8. The limitation of this study is that the media is obtained by the relatively acute response of lung fibroblast against asbestos fibers. Importantly, further study with different settings including long term asbestos exposure, and various asbestos will give more insight into the asbestos-related tumor microenvironment.

## Supporting information

S1 Fig SEM images of three types of asbestos. Chrysotile of serpentine group features bundled fibrils. (A) Chrysotile (B) Amosite and (C) Crocidolite of amphiboles group are needle-like in shape.
(TIF)

S2 Fig X-ray diffraction (XRD) patterns of three types of asbestos. (A) Chrysotile, (B) Amosite, and (C) Crocidolite. Their crystallographic information was identified using XRD and checked using Joint Committee on Powder Diffraction Standards.
(TIF)

S3 Fig Confocal laser microscopy of IMR-90 cells exposed to asbestos fibers. Cells were treated with chrysotile, amosite, or crocidolite (50 mg/L) for the indicated times and stained to visualize the mitochondria (red), filamentous actin (green), and nuclei (blue).
(TIFF)

S4 Fig Optimizing lung cancer cell number. Lung cancer cells were seeded onto E-plate 16 from cell densities of 5,000 cells/well to 60,000 cells/well. Media derived from lung fibroblasts were added. The optimum number of (A) NCI-H358 (B) Calu-3, and (C) A549 cells were 10,000 cells/well, 40,000 cells/well, and 4,000 cells/well, respectively.
(TIF)

S5 Fig Viability of IMR-90 cells treated with asbestos, H_2_O_2,_ and UV. ATP production of viable cells was determined using the CellTiter-Glo luminescence assay (Promega, Southampton, UK). (A) Viability of IMR-90 cells exposed to 50 mg/L asbestos (chrysotile, amosite, and crocidolite) for 24 h. (B) Viability of 24 h-cultured IMR-90 cells after exposure to 0.01, 0.1, 1, and 10 mM H_2_O_2_ for 3 h. (C) Viability of 24 h-cultured IMR-90 cells after UV irradiation (10, 25, 50, and 100 J/m^2^).
(TIF)

S6 Fig Titration of lung cancer cells for migration in RTCA. (A) NCI-H358 and (B) Calu-3 cells could not migrate toward CIM-plate 16. (C) A549 cells showed different rates of migration according to the cell seeding numbers.
(TIF)
