# Supplementary figures and images for: Conditioned medium from asbestos-exposed fibroblasts affects proliferation and invasion of lung cancer cell lines
Source: PLoS One. 2019 Sep 6;14(9):e0222160. doi: 10.1371/journal.pone.0222160 (PMC6730856; doi:10.1371/journal.pone.0222160)

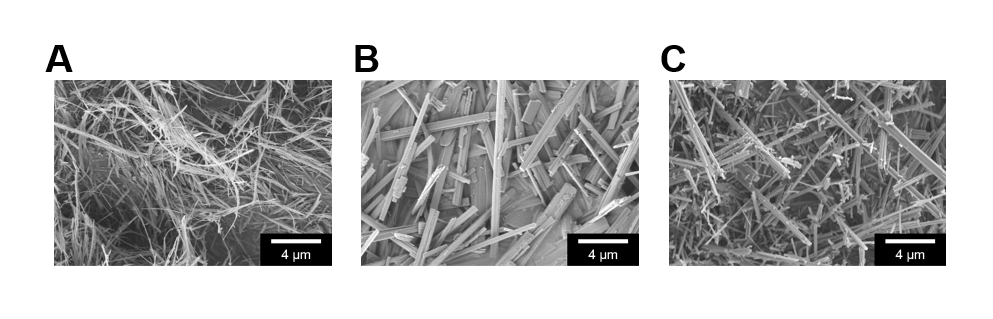

Supplement: S1 Fig — Chrysotile of serpentine group features bundled fibrils. (A) Chrysotile (B) Amosite and (C) Crocidolite of amphiboles group are needle-like in shape. (TIF) [file pone.0222160.s001.tif]

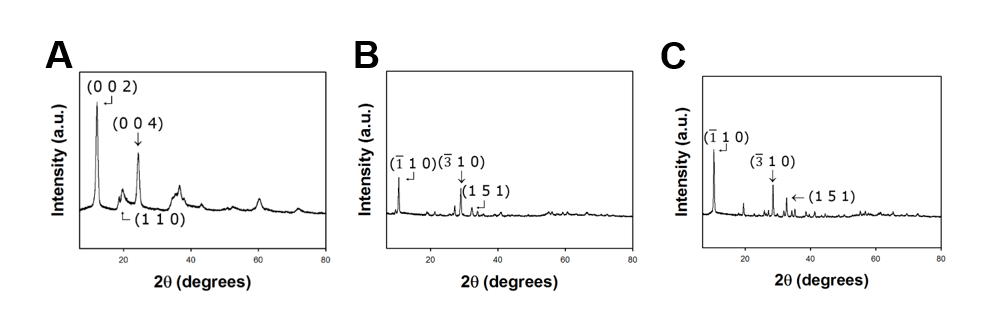

Supplement: S2 Fig — (A) Chrysotile, (B) Amosite, and (C) Crocidolite. Their crystallographic information was identified using XRD and checked using Joint Committee on Powder Diffraction Standards. (TIF) [file pone.0222160.s002.tif]

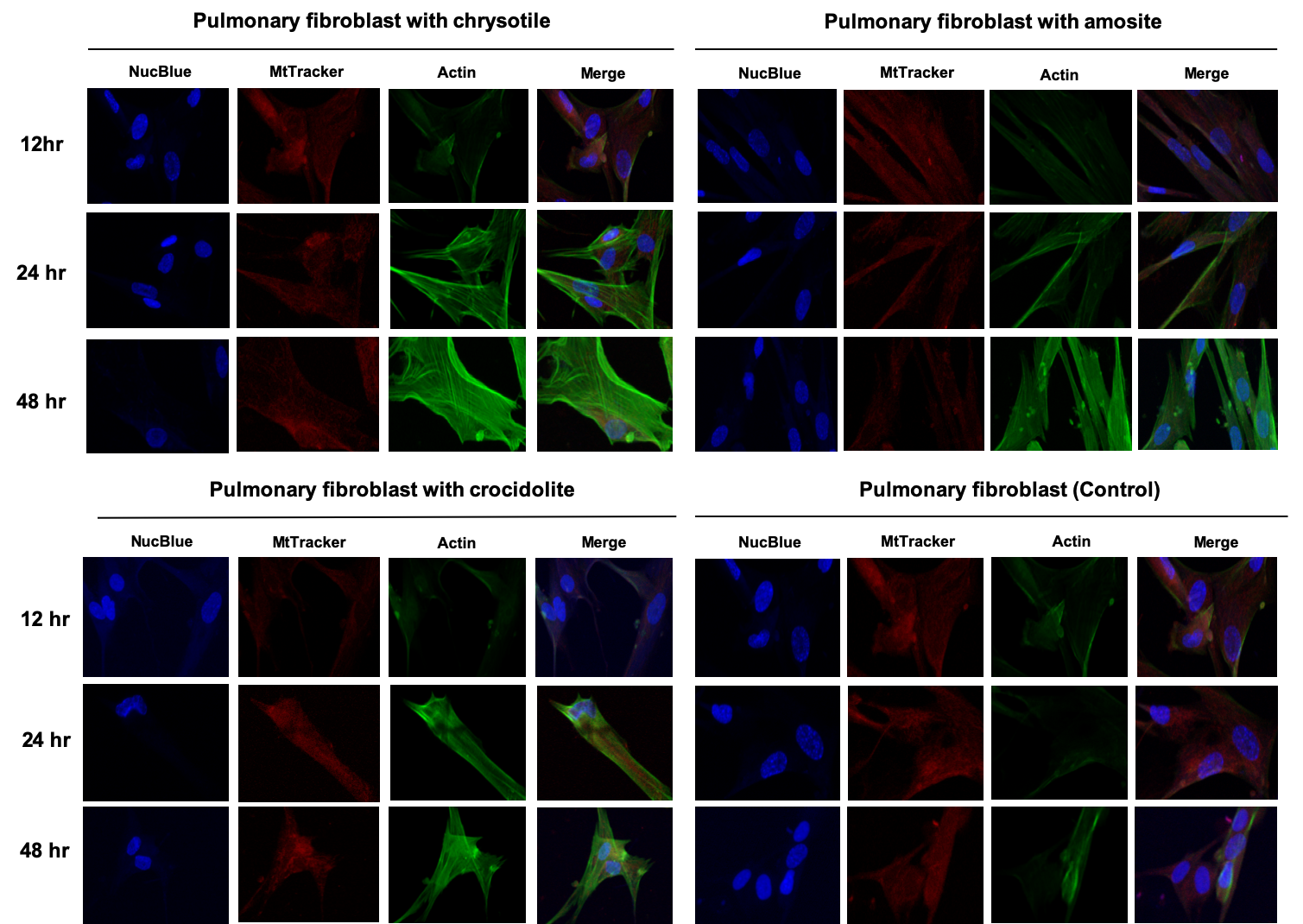

Supplement: S3 Fig — Cells were treated with chrysotile, amosite, or crocidolite (50 mg/L) for the indicated times and stained to visualize the mitochondria (red), filamentous actin (green), and nuclei (blue). (TIFF) [file pone.0222160.s003.tiff]

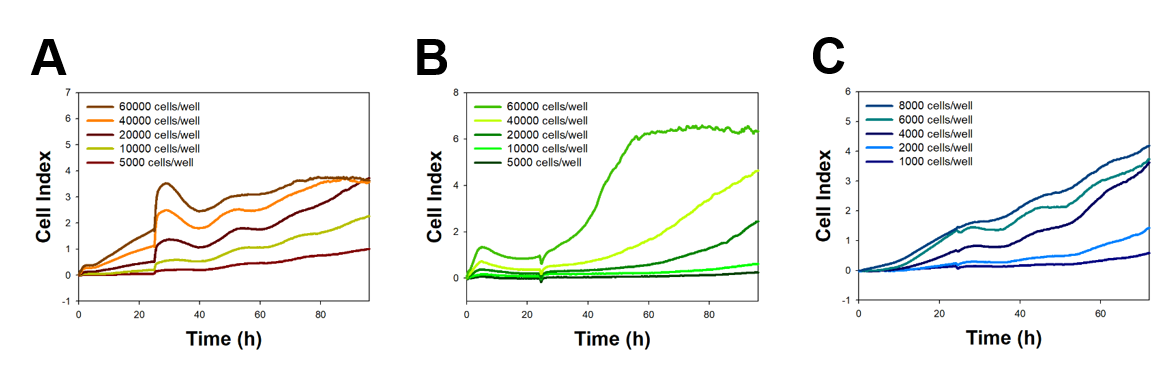

Supplement: S4 Fig — Lung cancer cells were seeded onto E-plate 16 from cell densities of 5,000 cells/well to 60,000 cells/well. Media derived from lung fibroblasts were added. The optimum number of (A) NCI-H358 (B) Calu-3, and (C) A549 cells were 10,000 cells/well, 40,000 cells/well, and 4,000 cells/well, respectively. (TIF) [file pone.0222160.s004.tif]

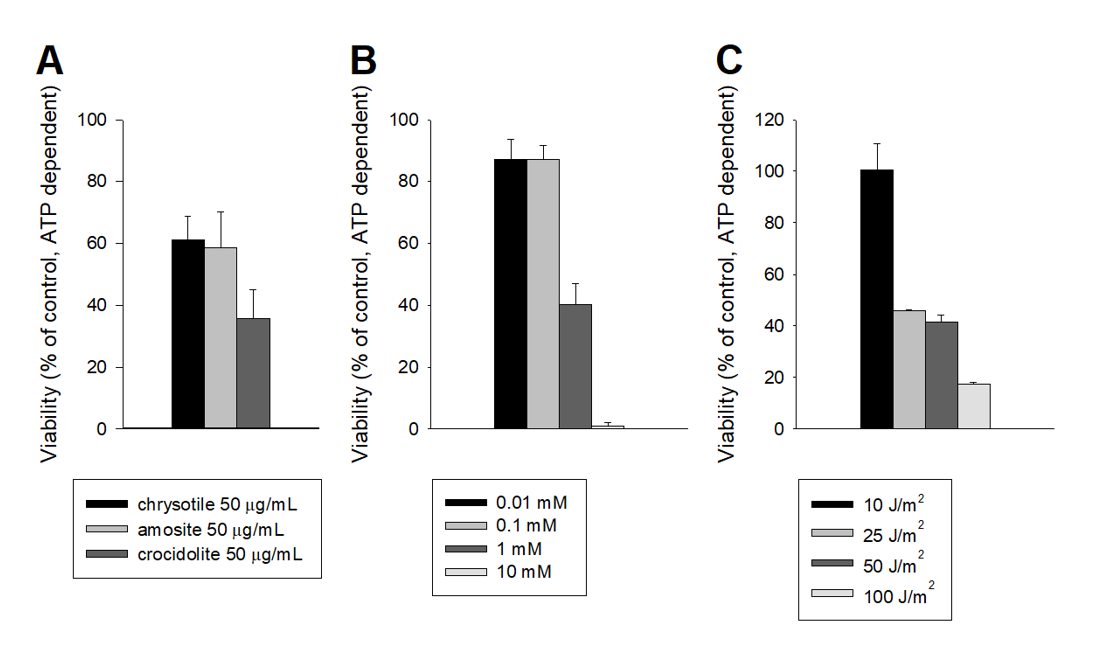

Supplement: S5 Fig — ATP production of viable cells was determined using the CellTiter-Glo luminescence assay (Promega, Southampton, UK). (A) Viability of IMR-90 cells exposed to 50 mg/L asbestos (chrysotile, amosite, and crocidolite) for 24 h. (B) Viability of 24 h-cultured IMR-90 cells after exposure to 0.01, 0.1, 1, and 10 mM H2O2 for 3 h. (C) Viability of 24 h-cultured IMR-90 cells after UV irradiation (10, 25, 50, and 100 J/m2). (TIF) [file pone.0222160.s005.tif]

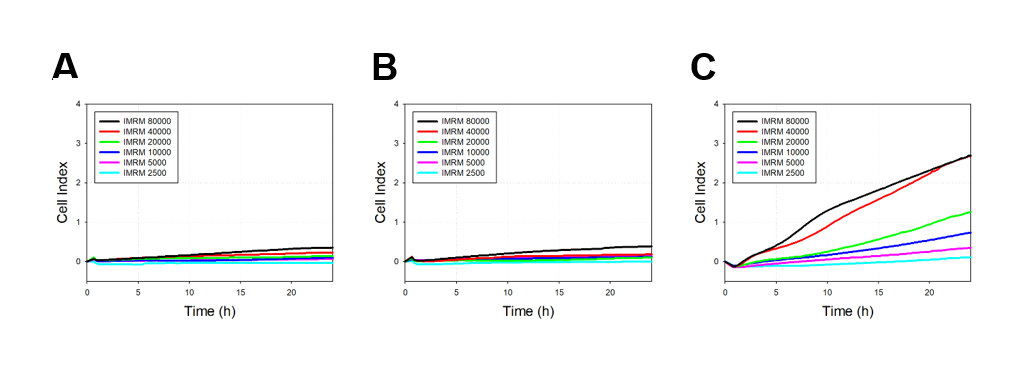

Supplement: S6 Fig — (A) NCI-H358 and (B) Calu-3 cells could not migrate toward CIM-plate 16. (C) A549 cells showed different rates of migration according to the cell seeding numbers. (TIF) [file pone.0222160.s006.tif]
